# Co-Delivery of siRNA and Docetaxel to Cancer Cells by NLC for Therapy

**DOI:** 10.1021/acsomega.3c09098

**Published:** 2024-02-27

**Authors:** Behiye Şenel, Ebru Başaran, Evrim Akyıl, Umay Merve Güven, Gülay Büyükköroğlu

**Affiliations:** † Faculty of Pharmacy, Department of Pharmaceutical Biotechnology, Anadolu University, 26470 Eskisehir, Türkiye; ‡ Faculty of Pharmacy, Department of Pharmaceutical Technology, Anadolu University, 26470 Eskisehir, Türkiye; § Faculty of Pharmacy, Department of Pharmaceutical Technology, Cukurova University, 01330 Adana, Türkiye

## Abstract

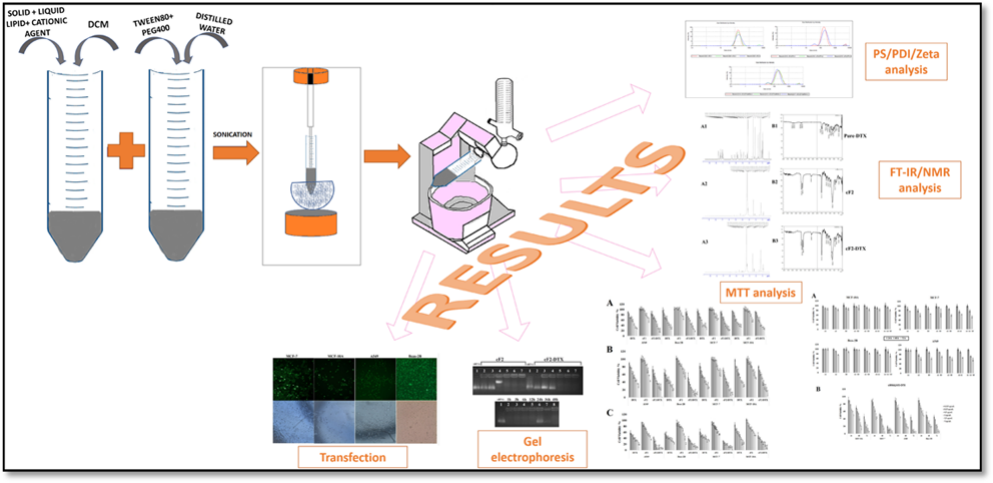

The present study aims to develop a delivery system that can carry small interference RNA (siRNA) with small-molecule chemotherapeutic drugs, which can be used in cancer treatment. The drug delivery system combines the advantages of a therapeutic agent with two different mechanisms to ensure that it is used efficiently for cancer therapy. In this study, a nanostructured lipid carrier system was prepared, Docetaxel was loaded to these systems, and the Eph siRNA was adsorbed to the outer surface. In addition, DOTAP was added to the lipophilic phase to load a positive charge on the lipidic structure for interaction with the cells. Moreover, characterization, cytotoxicity, and transfection procedures were performed on the whole system. This candidate system was also compared to Taxotere, which is the first approved Docetaxel-containing drug on the market. Given the results, it was determined that the particle size of NLC-DTX was 165.3 ± 3.5 nm, the ζ potential value was 38.2 ± 1.7 mV, and the PDI was 0.187 ± 0.024. Entrapment efficacy of nanoparticles was found to be 92.89 ± 0.21%. It was determined that the lipidic system prepared *in vitro* release analyses were able to provide sustained release and exhibit cytotoxicity, even at doses lower than the dose used for Taxotere. The formulations prepared had a higher level of effect on cells when compared with pure DTX and Taxotere, but they also exhibited time-dependent cytotoxicity. It was also observed that the use of Eph siRNA together with the chemotherapeutic agent *via* formulation also contributed to this cell death. The results of the present study indicate that there is a promising carrier system in order to deliver hydrophilic nucleic acids, such as siRNA, together with lipophilic drugs in cancer treatment.

## Introduction

When a single cell begins to divide without any regulatory mechanism, a cell colony forms that interferes with normal physiological functions. Cancer develops as a result of this process. Cancer is the leading and increasing cause of death throughout the world.^1^ Chemotherapy is still the most commonly used treatment approach in cancer treatments, followed by radiotherapy, surgery, hormone treatments, biological treatments, and targeted treatment approaches.^2^ The only key to successful cancer treatment is treatment regimens in which multiple treatment agents are used in combination. The aim of this combined treatment is to increase the quality of life, control disease progression, and ensure long survival without excessive toxicity. Such treatment involves the use of different small-molecule cytotoxic drugs or the combination of small-molecule chemotherapeutic drugs together with nucleic acid molecules or monoclonal antibodies.^3^

Docetaxel (DTX), a substance obtained from the needle leaves of the yew tree, is the second-most-important drug in the group of drugs called taxoids used in cancer treatment. DTX creates its antitumor effects by causing microtubule stabilization during the formation of the mitotic spindle in the cell, stopping cell division, and causing the cell to undergo apoptosis. DTX is taken into the cell faster and stays in the cell longer when compared with other taxane compounds. Thanks to these properties, it was shown to be 2–4 times more potent than docetaxel in *in vivo* and *in vitro* antitumor activity studies.^4,5^

In recent years, thanks to the advancements in technology, silencing or increasing unwanted gene expression using nucleic acid therapeutics (*e.g.*, siRNA, shRNA, CRISPR7/Cas9, and antisense oligonucleotides) became an extremely popular method to prevent tumor cell growth and invasion.^6^ The molecules included in these nucleic acid therapeutics, which are called RNA interference (RNAi), constitute a natural defense mechanism for the invasion of exogenous genes. siRNA and miRNAs, which can eliminate the expression of target genes in a sequence-specific manner by allowing for targeted mRNA degradation or suppression of mRNA translation, are important components of the RNAi system. The main difference between them is that siRNA can typically trigger more efficient and specific gene silencing than miRNA, whereas miRNA can simultaneously stop the expression of several different target genes. Therefore, siRNA and miRNA play different roles in pharmaceutical applications.^7^ Although many antisense oligonucleotide products have been discovered for use in cancer treatments, there was no commercially available siRNA/miRNA product until 2018. The United States Food and Drug Administration (FDA) and the European Commission (EC) approved the first siRNA product ONPATTRO (Patisiran), which will be used in the treatment of hereditary amyloidogenic transthyretin amyloidosis (hATTR) in adults with polyneuropathy in 2018. Then, GIVLAARI (Givosiran) was approved for the treatment of adults with acute hepatic porphyria (AHP) in 2019, followed by OXLUMO (Lumasiran) approved for the treatment of primary hyperoxaluria type 1 (PH1) in pediatric and adult subjects in 2020, LEQVIO (Inclisiran) approved for the treatment of heterozygous familial hypercholesterolemia (HeFH) or clinical atherosclerotic cardiovascular disease (ASCVD) in 2021, and AMVUTTRA (Vutrisiran) approved for the treatment of hereditary amyloidogenic transthyretin amyloidosis (hATTR) in adults with polyneuropathy in 2022.^8^

Eph receptors (erythropoietin-producing hepatoma) were selected as the siRNA target. Eph receptors are the largest receptor member of the Tyrosine Kinaz (TFK) family, which can carry signals from the outer environment to internal compartments, thus directly affecting direct gene transcription or indirectly affecting secondary messenger production. Eph A and B classes are divided into various groups. For example, the EphA class consists of EphA1-A8 and EphA10 members, which are randomly connected to five ephrin molecules (EphrinA1-A5). EphB is a class consisting of EphB1-B4 and B6 connected to the ligand of three EphrinBs (EphrinB1-B3).^9,10^

The present study aims to prepare nanostructured lipid carrier (NLC) systems and investigate their effects on cells after loading genetic material and a chemotherapeutic agent into this system. For this reason, a nanostructured lipid carrier system was prepared with solid lipid and liquid lipid, then DTX was loaded into the system, and siRNA was adsorbed to the outer surface. Then, *in vitro* characterization, release property study, and cytotoxicity analyses were performed.

## Experimental Section

### Materials

Labrafac lipophile WL 1349 and Dynasan 116 were donated by Gattefossé (Lyon, France). siRNA EphA1, EphA2, EphB3, and Control siRNA-FITC were both provided by Santa Cruz Biotechnology (Freiburg, Germany). Docetaxel and DOTAP, MTT dye, and dimethyl sulfoxide (DMSO) were obtained from Sigma-Aldrich (Steinheim, Germany). Taxotere (20 mg/mL) was purchased from a pharmacy. Ultrapure water was obtained by using a Milli-Q system (Millipore, Bedford, MO). All of the chemicals were of analytical grade.

### Preparation of the NLCs

NLCs were prepared by using the sonication-evaporation method with some modifications.^11^ In this method, the amounts of solid lipid (Dynasan 116) and liquid lipid (Labrafac lipophile WL 1349) were weighed (Table 1), and they were dissolved in 500 μL of dichloromethane (DCM) in a falcon tube. Distilled water and surfactants (T; Tween 80 and P; PEG400) were weighed in another falcon tube and gently vortexed to mix homogeneously. The two liquid phases were immediately dispersed within each other and sonicated at 35% power for 5 min. Meanwhile, to prevent the prepared formulation from getting too hot and being affected by DCM and sonication, it was occasionally dipped into a beaker filled with cold water, preventing the system from overheating. At the end of sonication, the balloon was placed in a rotavapor and the organic solvent was allowed to evaporate. In the following days, it was checked whether there was phase separation or creaming. The cationic feature of the system was created by adding DOTAP (2 mg/mL) to the lipid phase.

**Table 1:** Ratios of Lipid (Solid/Liquid) and Surfactants (T; Tween 80 and P; PEG400) in Formulations

	F1	F2	F3	F4	F5	F6	F7	F8	F9	F10
lipids w/w% (solid/liquid)	4% (1:1)	4% (1:2)	4% (1:4)	4% (2:1)	4% (4:1)	4% (1:1)	4% (1:2)	4% (1:4)	4% (2:1)	4% (4:1)
surfactants w/w% (T/P)	4% (1:2)	4% (1:2)	4% (1:2)	4% (1:2)	4% (1:2)	4% (1:1)	4% (1:1)	4% (1:1)	4% (1:1)	4% (1:1)

### Preparation of the NLC-Loaded siRNA and DTX

To achieve the optimum formulation, 5 mg/mL DTX was added to the lipid phase and dissolved in DCM. Subsequently, the lipid phase substance was mixed with the liquid phase containing surface active agent with the same procedures as described above in the Preparation of the NLCs section.

The particle size and ζ potential of the formulation were measured after the organic solvent was evaporated. After the appropriate formulations were determined, siRNAs (EphA1, EphA2, and EphB3) selected as targeted genetic material were enabled to interact with cationic nanostructured lipid particles by electrostatic interaction. Accordingly, the amounts of siRNA calculated according to the examination made on formulations were taken in certain proportions and mixed carefully. Then, they were incubated at 37 °C for 20 min to allow electrostatic interaction. Whether there was an electrostatic interaction was determined by gel documentation.

### Characterization Analyses

#### Particle Size/Distribution and ζ Potential Measurement

Particle sizes and ζ potentials of the prepared formulations were measured by using the Malvern Zeta Sizer Nano ZS instrument (25 ± 2 °C), and the measurement was repeated three times for each sample.^11^

### NMR and FT-IR Analyses

For FT-IR analyses, the samples were first lyophilized. Then, first, KBr disks were prepared for DTX, docetaxel-loaded NLCs, and empty NLCs, and then the FT-IR spectrum was detected in the wavelength range of 4000–500 cm^–1^ by using the FT-IR (Shimadzu IR Prestige-21, Japan) device. DTX, DTX-loaded NLCs, and empty NLCs were dissolved in deuterated DMSO (DMSO-*d*_6_) for NMR (^1^H NMR) analysis in the formulations. Analyses of the samples were performed by using an NMR (Bruker 500 MHz UltraShield NMR, Germany) device.^12^

### Gel Documentation Analyses

These analyses were performed to determine the genetic material binding rates of the prepared nanoparticles and their protection properties in the presence of enzymes such as DNAz-RNAz. In order to determine the binding rates, the particles and genetic material were first subjected to an electrostatic interaction at room temperature. After being exposed to electrostatic interaction in the presence of serum for its protection properties, they were kept in a medium containing 20% fetal bovine serum for 1, 3, 6, 12, 24, 36, and 48 h. Then, the samples were taken at the end of the period. The samples were mixed with a loading buffer in both procedures. Then, they were applied to the wells on the 1% agarose gel and run in an electrical field at 50 mV for 30 min. After execution, the gel was visualized under UV light (UViTec Alliance 4.7, Cambridge, U.K.).^11^

### *In Vitro* Loading Capacity and Drug/Genetic Material Release Analysis

The free DTX content in the solution containing NLCs was calculated to determine the entrapment efficiency (EE%).^13^ For this purpose, first, the solution was centrifuged at 16,400 rpm for 45 min. After that, the DTX content of NPs was assessed by extracting DTX from the nanoparticles. 500 μL of NLC formulations was taken, 1.5 mL of DCM was added, and the mixture was vortexed to dissolve the particles in the organic phase. This solution containing a dichloromethane phase was poured into a quartz cuvette, and the spectrum was then taken. These procedures were repeated four times with a UV–visible spectrophotometer (Shimadzu UV–vis 160, Kyoto, Japan) at 230 nm.

The EE of nanoparticles was calculated from eq 1.
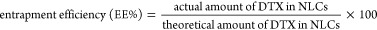
1

The release behaviors of DTX and genetic materials from formulations were investigated in this analysis.^13^ In the release procedure, pH 7.4 and 5.0 phosphate buffers (PBS) containing 0.5% Tween 80 were used as the dialysis bag method and release medium. At 37 ± 0.5 °C, a 1000 μL formulation containing the amount of substance to be determined during formulation preparation, providing sink conditions, was placed in the dialysis membrane (MWCO: 14,000 Da) with the capability to pass the substance with a certain molecular weight. Before using the membrane, it was kept in a release medium for 2 h. The dialysis bags containing formulations and Taxotere were immersed in 100 mL of PBS (pH 7.4), and they were then placed on a magnetic stirrer and stirred with the membrane at 150 rpm. At predetermined time intervals (0th, 1st, 3rd, 6th, 12th, 24th, 48th, 72nd, 96th, 120th, 144th, and 192nd hours), 500 μm of the solution was taken and an equal volume of fresh medium was supplied. After samples were taken, 1.5 mL of dichloromethane was added; while the DTX remained in the organic phase, the siRNA remained in the water-containing phase. After the organic phase and water phase were carefully separated by using a pipet, a quantification procedure was conducted using UV analysis *via* a UV spectrophotometer for Docetaxel and NanoDrop for siRNA. The percent cumulative amount of DTX and siRNA released from NLCs was calculated. The results were compared to Taxotere.

### *In Vitro* Studies

#### Cell Culture

The effects of the NLC formulations prepared on the cells were examined. Beas-2B, A549, MCF-10A, and MCF-7 cells were used in these studies. These cells were chosen to determine the effects of both Docetaxel and siRNA-EpH on different cell lines.

### Cell Viability Analyses

Cytotoxic effects of NLCs on different cell lines were assessed by using a tetrazolium salt reduction (MTT) assay, which is frequently used in the laboratory where the present study was carried out. Briefly, all of the cell lines were grown in Dulbecco’s modified Eagle’s medium (DMEM) supplemented by 10% fetal bovine serum (FBS), and penicillin (100 units/mL) and seeded onto a 96-well plate at a density of 1 × 10^4^ cells per well. The cells were incubated at 37 °C for 24 h under a humidified atmosphere containing 5% CO_2_. After 24 h of incubation time, the growth medium was discharged and added with fresh growth medium containing different concentrations of formulations, Taxotere and DTX only. The plates were put into an incubator for 24, 48, and 72 h. After incubation time intervals, the culture medium was withdrawn and 25 μL of MTT solution (in 5 mg/mL PBS buffer) was added and the plates were incubated at the same conditions for another 4 h. Then, 200 μL of analytical-grade DMSO was added to each well to dissolve the formazan crystals. Following 30 min of incubation, absorbances of the plates were measured at 570 nm using a spectrometric microplate reader (Cytation 5 Cell Imaging Multi-Mode Reader, U.K.) 16,19. The results were expressed as the percentage of absorbance of control cells with no treatment.^14^

### Transfection Studies

Cells were seeded in 96-well plates at 15,000 cells per well in FBS-free DMEM 24 h before transfection experiments. Considering MTT and siRNA binding studies from the prepared formulation, electrostatic interaction was applied with 2 μL of cF2-DTX formulation and 0.2 μL of FITC conjugated siRNA (Santa Cruz Biotechnology, sc-36869). After the electrostatic interaction, the complexes were applied to the wells, and 6 h later, 20% FBS was added and incubated for an additional 24 h. After the incubation period, the media containing the complexes were replaced with fresh medium. The images were examined under a fluorescent microscope (Leica DM 4000) and fluorescent images were taken. In addition to fluorescence microscopy analysis, approximately 10000 cells (transfected and nontransfected) were counted in each 20 different areas of the wells and the transfectıon index (TI) was calculated using the equation below.
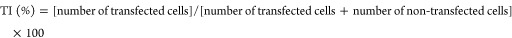
2

### Statistical Analyses

The MTT assay data is expressed as the mean ± standard deviation on the graphic. The differences between the treatment and control groups were analyzed considering the concentration-effect-time curves by using linear regression analysis with Minitab 18. The other differences were analyzed by using the variance analysis and Student’s *t* test. Statistical significance was set at *p* < 0.05.

## Results and Discussion

Research and development of safer and more effective alternative delivery systems for drugs with poor water solubility in the pharmaceutical industry is of great importance. In general, the commercially available options include soft gelatin capsules, tablets, or oral suspensions in the markets for such preparations.^15^ Docetaxel (DTX), a second-generation taxane group, is considered one of the most potent chemotherapeutic agents in clinical setting. It is almost insoluble in water, as other taxane compounds.^16^ DTX is mainly used in the treatment of cancer types, which have not been successfully treated with anthracycline-based chemotherapy, including breast, prostate, and non-small-cell lung cancer. Additionally, clinical findings showed that DTX has cytotoxic activity against colorectal, ovary, liver, renal, gastric, and head and neck cancers, and melanoma.^17^ Previous studies reported that DTX is more advantageous than Paclitaxel (PTX), which is the first drug of the taxane group. *In vitro* studies revealed that DTX has 2.5 times more potential in inhibiting cell proliferation when compared to PTX. This is partly because DTX can inhibit cell mitosis in both S and G2/M phases, whereas PTX can inhibit cell mitosis only in the G2/M phase. In addition, DTX has a longer retention time in tumor cells in comparison to PTX due to its better uptake into cells, slower elimination from cells, and longer terminal elimination half-life from tumor tissue.^18^ The only drawback of Taxotere, the only DTX product in the market, seems to be that it contains a high Tween 80 and is applied with alcohol. There is information that a high Tween 80 content causes more side effects and hypersensitivity. Moreover, alcohol application is unfortunately not suitable for some ethnicities due to religious beliefs. Besides that, it was also stated in the literature that some signs indicating the beginning of apoptosis are observed in the cells after ethanol application in breast cancer; however, the apoptosis is not complete and does not occur at a sufficient level. It was stated that inefficient initiation of apoptosis causes cancer cells to not be adequately affected and the cells to be restored, or it causes an increase in the recurrence of cancer since the cells gain a more aggressive phenotype as a result of irregular gene amplification and DNA damage in the slightly affected cells.^19,20^ Therefore, this study aims to monitor the effects of decreased doses of DTX with Eph siRNA on cancer cells with new delivery systems. It is aimed to keep water-insoluble DTX in the oil phase and to sustain the effect by adding the hydrophilic Eph siRNA to the water phase.

Nanostructured lipid carrier (NLC) systems are second-generation innovative lipid nanoparticles that serve as bioactive carrier systems. NLCs, composed of a mixture of liquid and solid lipids, have been developed to overcome some of the potential limitations of the solid lipid nanoparticle.^21^ The main advantages of NLTs include improving the stability and drug loading capacity and preventing drug curtailment during storage. For this reason, in this study, NLC systems were developed by using solid and liquid lipids together instead of using only solid lipids.^22^ In this study, it was aimed to develop new systems for DTX and siRNA delivery by making use of the advantages of these systems and to monitor the effects of decreased doses of DTX with Eph siRNA on cancer cells using new delivery systems. So, it was planned to keep the water-insoluble DTX in the oil phase and to sustain the effect by adding the hydrophilic Eph siRNA to the water phase. After the formulations were prepared by the sonication and solvent evaporation method, first of all, characterization analyses were carried out and the formulation with optimum properties was selected. The resulting DTX-loaded NLC formulations were found to be in a milky emulsion with a full white color. In characterization studies, it was evaluated first whether there is any creaming/phase separation occurred during the formulation preparation phase or during waiting 1-week waiting period. Then, particle size and ζ potential analyses were examined in the formulations that were found to be homogeneous.

### Particle Size and Polydispersity Index

**Figure 1:**
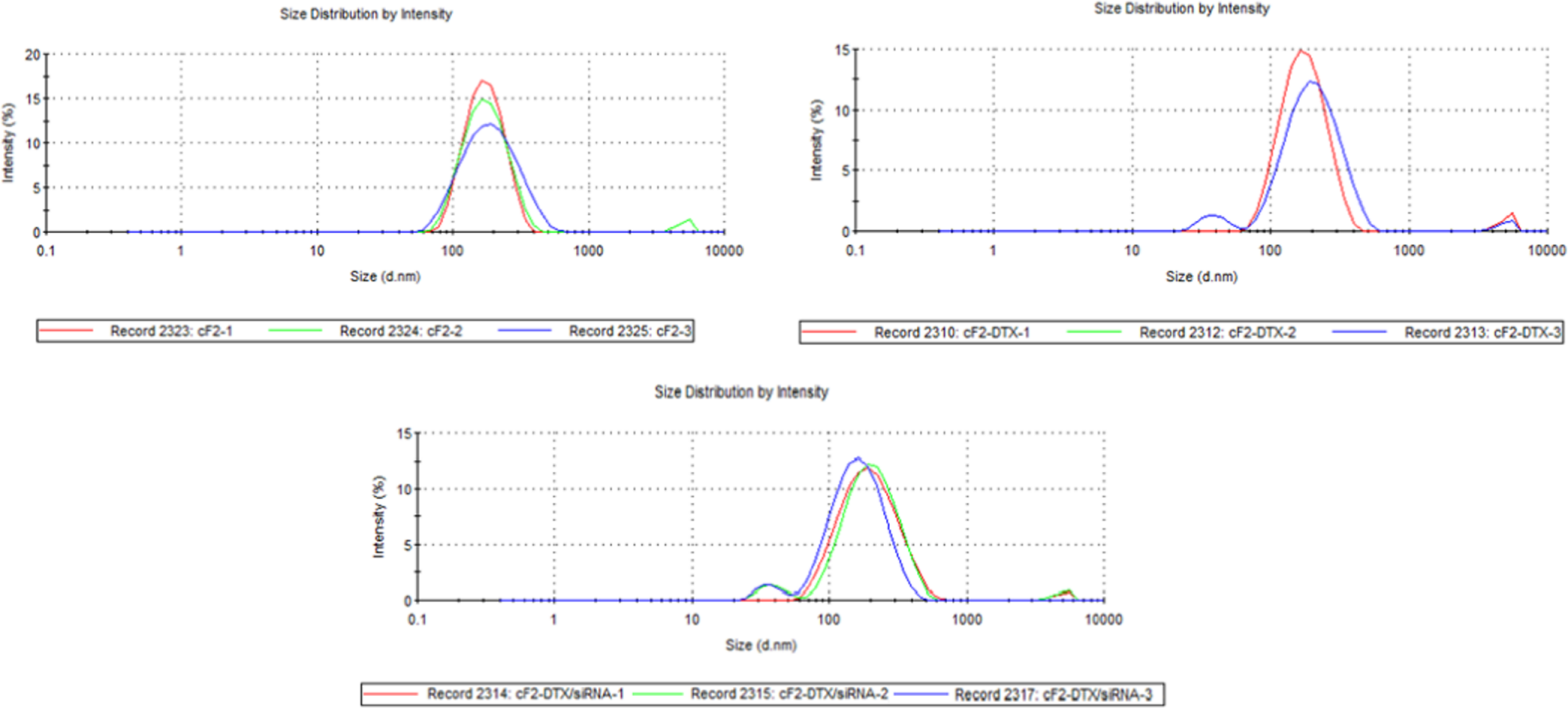
Particle size distribution by intensity of cF2, Cf2-DTX, and cF2-DTX/siRNA (*n* = 3)

The formulations were divided into three groups as formulations containing no cationic agent or DTX (F), those containing only DTX (F-DTX), and those containing both cationic agent and DTX (cF-DTX), and they were analyzed. Since creaming was observed during preparation in the F4, F5, F7, F9, and F10 formulations, no cationic agent or DTX was loaded, and the process was continued with other formulations. Accordingly, the results are listed in Table 2 and Figure 1, respectively. Additionally, the pH values of the prepared formulations were found to range between 6.5 and 6.8.

**Figure 2:**
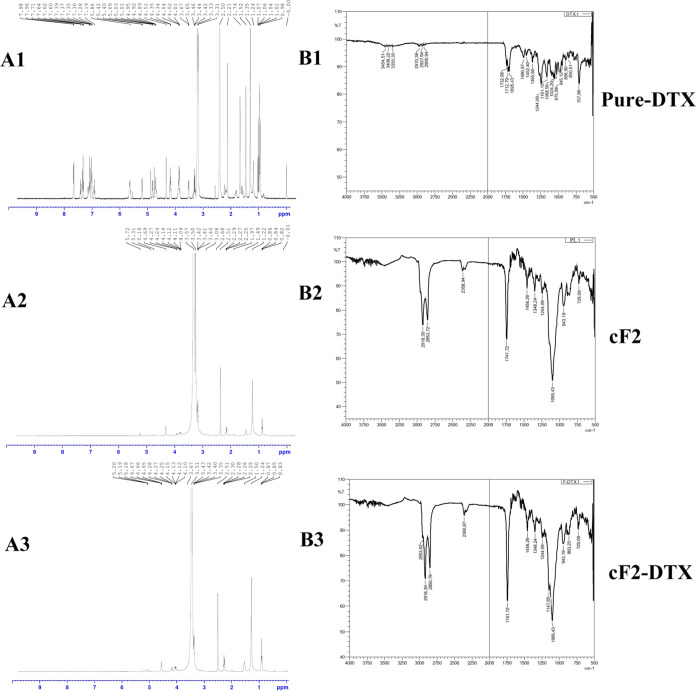
Results of ^1^H NMR (A1–A3) and FT-IR (B1–B3) analyses of pure DTX, cF2, and cF-DTX formulations.

**Table 2:** Results of Particle Size, PDI, and ζ Potential Results in Prepared Formulations ± SD

	particle size (nm)	PDI	ζ potential (mV)
F1	218.2 ± 1.6	0.222 ± 0.035	–7.77 ± 3.5
cF1	225.5 ± 5.5	0.241 ± 0.014	27.62 ± 3.2
cF1-DTX	200.4 ± 3.3	0.198 ± 0.022	25.1 ± 2.0
F2	140.2 ± 1.4	0.198 ± 0.009	–8.78 ± 2.2
cF2	145.1 ± 2.3	0.227 ± 0.005	39.6 ± 1.2
cF2-DTX	165.3 ± 3.5	0.187 ± 0.024	38.2 ± 1.7
F3	237 ± 3.8	0.155 ± 0.036	–9.7 ± 2.1
cF3	265.4 ± 3.1	0.174 ± 0.047	16.2 ± 3.3
cF3-DTX	288.6 ± 4.6	0.333 ± 0.041	11.4 ± 2.1
F6	400.6 ± 5.2	0.268 ± 0.034	–5.65 ± 2.9
cF6	436.4 ± 3.2	0.253 ± 0.012	–6.35 ± 3.5
cF6-DTX	455.65 ± 3.8	0.467 ± 0.054	+15.6 ± 2.5
F8	444.8 ± 6.8	0.555 ± 0.039	–9.65 ± 2.0
cF8	467.5 ± 3.4	0.423 ± 0.023	–10.3 ± 3.0
cF8-DTX	480.4 ± 4.2	0.360 ± 0.054	+12.7 ± 2.1
c-F2-DTX/siRNA	170.65 ± 3.6	0.123 ± 0.011	31.2 ± 2.2

In the formulations, the particle size varied between 140.2 ± 1.4 and 480.4 ± 4.2, and the polydispersity index varied between 0.155 ± 0.036 and 0.555 ± 0.039. No statistically significant difference was observed between formulations with active ingredients and those without any (*p* > 0.05). It was observed in the analyses that creaming occurred with the increase of lipid ratio in the formulation or phase separation occurred over time. In particular, it was found that the particle size decreased when the lipid mixture was kept low and the amount of solid lipid was lower than that of liquid lipid. Therefore, it is thought that the addition of the liquid lipid may be effective on the particle size, especially in lipidic formulas. As with NLCs, it can be said that smaller particles are obtained by using liquid lipids in general and reducing the viscosity of the oil phase.^23−25^ However, it was determined that increasing the PEG400 concentration in surfactants reduces the particle size. It was predicted that this result might be due to the higher viscosity of PEG400 in comparison to Tween 80, and such results were also encountered in the literature.^26,27^ The use of heterogeneous lipids in lipidic formulations instead of homogeneous ones (such as pure saturated fatty acid or triglycerides) can lead to the formation of smaller-sized particles. NLCs also provide long-term active stability during storage, as the presence of the liquid lipid in the carrier particle reduces the amount of active crystallization. NLC formulations require less surfactant than liquid emulsion systems, which allows for more active agent loading.^28,29^

Another important characteristic feature is the PDI value. This parameter indicates the homogeneity and size distribution of the particles. Homogeneous and monodisperse particles have low PDI values in the range of 0.1–0.25. Moreover, the PDI values higher than 0.5 indicate high polydispersity and wide distribution.^30^ In the formulas prepared in this study, only the F1 and F3 formulations were found to have PDI values lower than 0.25, whereas the others had values lower than 0.5. Therefore, it can be said that they show a monodisperse and homogeneous distribution.

### ζ Potential

ζ Potential is used to describe the charge on the particles. The ζ potential helps to predict the stability and flocculation effect in emulsion systems. If the ζ potential decreases below a certain level, the colloid will precipitate due to attractive forces. Conversely, a high ζ potential (>|30| mV) offers an advantage in creating a stable system.^31−34^ As shown in Table 2, ζ potential values were found to be negative in systems without cationic agent as expected (between |−11.2| and |−5.65| mV), whereas ζ potentials of formulas increased up to +38.2, especially for F2 formulation, after the addition of DOTAP.

In general, lipid nanoparticles are negatively charged on their surface due to the carboxyl groups (COO^®^) present in their composition. Therefore, the ζ potential values of all formulations prepared without adding any cationic agent are considered negative. Moreover, Tween 80, a surfactant, is a nonionic surfactant and has no effect on the ζ potential. Even though it is seen in the literature that ζ potential values of formulations decrease with increasing liquid–solid lipid ratio, there is no significant correlation in long-term stability studies.^34,35^

It was stated in other studies that the presence of a solid lipid reduces the ζ potential values. It was also reported that there are ζ levels higher than SLNs, especially in preparations in the form of emulsions prepared with liquid lipids.^36−38^

In the present study, it was observed that the ζ potential value increased, especially when the liquid lipid ratio increased in the formulation. The highest ζ potential value was observed in formulations F1 and F2. A positive but relatively low ζ potential was also observed in samples F3, F6, and F8. Since it was thought that these formulas could not bind to siRNA sufficiently, they were excluded from the study. The formulations F1 and F2 were also evaluated in terms of ζ potential and particle size, and it was decided to continue with formulation F2.

### FT-IR and ^1^H NMR Analyses

FT-IR and ^1^H NMR analyses were performed to determine whether there was any intramolecular interaction between DTX and the lipid structures in the formulation (Figure 2).

In FT-IR analyses of pure DTX, characteristic bands were detected at frequencies of 1732.08, 1712.79, and 1695.43 cm^–1^ (C=O stretching), 1452.40 cm^–1^ (C=C stretching), 1244.09 cm^–1^, and 1161.15 cm^–1^ (C–N stretching), whereas not very sharp bands were detected between the frequencies of 2900 and 3400 cm^–1^ (C–H stretching). A very sharp fingerprint region (C–O stretching) was detected in formulation cF2 at 1099.43 cm^–1^ (C–O stretching), and sharp bands were detected at 1741.72 cm^–1^ (C=O stretching), 2852.72 cm^–1^, and 2918.30 cm^–1^ (C–H stretching). The spectra of cF2-DTX were analyzed very similar to those of cF2. It was observed that the characteristic peaks seen only in DTX FT-IR analyses were not observed in the cF2-DTX formulation. The reasons for this are the low amount of DTX loaded into the formulation and the possibility of different physical–chemical interactions due to the formation of various bonds between NLC components and DTX. It is thought that these interactions and their small amount make it difficult to detect bands and monitor frequency changes. However, the similarity of the characteristic bands seen in cF2 and cF2-DTX indicates that NLC formulation can be prepared without any chemical interaction.^13,39−42^

Examining the NMR analysis results in pure DTX, very sharp −CH peaks were seen at 1.04, 1.06, 1.07, 1.35, 1.52, 1.74, 2.21, 2.50, 3.33, and 3.42 ppm and −OH groups (aliphatic) between 3.46 and 6 ppm, whereas lower-intensity peaks were detected between 7–8 ppm in the aromatic region. Given the analyses of the cF2 placebo formulation, there was a CH3 peak at 0.82, 0.84, and 0.86 ppm, but very severe characteristic peaks were observed at 1.22, 2.68, 3.50, 3.57, and 1.49 ppm, 2.25–2.29, 2.51, and 4.69 ppm with lower-intensity characteristic peaks. In the DTX-loaded cF2 formulation, there was a CH_3_ peak at 0.83, 0.85, and 0.87, while very severe characteristic peaks were detected at 1.24, 2.51, 3.51, 1.50, 2.25, 2.26, 2.28, 2.30, and 4.10–4.15 ppm, and 4.25–4.28, 4.65–4.67, and 5.18–5.20 ppm with lower-intensity characteristic peaks. Accordingly, although cF2 and DTX-loaded cF2 formulation peaks seem very similar to each other, the peaks at 2.50, 3–4, and 4–5 ppm could not be fully differentiated because of overlapping, and the peaks were detected in similar spectra. However, the characteristic peak of DTX in the 7–8 ppm range was not observed in the formulation. The absence of the majority of the various functional group peaks determined for DTX in the cF2-DTX formulation suggests that the formulation of Docetaxel was completely confined within the lipid structure, and such results were also reported in the literature.^12,43^

### DSC Analysis

Lipid crystallization plays a crucial role in the performance of NLC carriers because it has an important effect on the drug-loading capacity of lipid particles and the drug release from them. It should be considered that the application of liquid lipids or oil ingredients may reduce the melting point of the lipid nanoparticle, resulting in easy diffusion of the drug through the nanocarrier. From this perspective, the melting behavior of the lipid mixture may be an important factor because it can predict the persistence of the amount of drug present in the nanocarriers during storage and have an effect on the evaluation of the drug effect.^44^

The DSC technique measures heat loss or gain in samples as a function of temperature by examining the physical or chemical changes in samples.^45^

**Figure 3:**
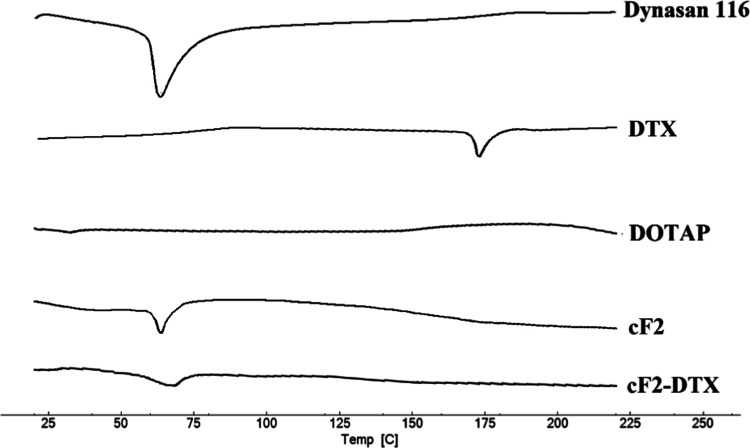
Differential scanning calorimetry (DSC) thermograms of Dynasan 116, DTX, DOTAP, cF2, and cF2-DTX.

For this reason, thermal analysis was performed to examine the crystal structure in the formulations and to elucidate the lipid structure. Since the changes that may occur in endothermic and exothermic peaks can provide information about the formulation stability, these peaks were examined. Thermograms of the components used and the formulation are shown in Figure 3, and thermal data are presented in Table 3.

**Table 3:** Thermal Data of Component and Formulation

component	*T*_peak_ (°C)	Δ*H* (J/g)
Dynasan 116	63.42	135.05
DTX	174.1	22.65
DOTAP	not determined	not determined
c-F2	65.1	32.40
cF2-DTX	70.3	15.1

Sharp endothermic melting peaks of pure Dynasan 116 at 63.42 °C and DTX at 174.1 °C were detected in crystalline form. No melting peak was observed for DOTAP. PEG400, Tween 80, and Labrafac lipophile WL 1349 are in liquid form at room temperature (initial analysis temperature), and therefore, it was not possible to visualize their melting points in DSC. The crystalline state of the lipid core is essential for lipophilic drug incorporation and sustained release properties of lipid nanoparticles. This endothermic peak of DTX was no longer seen in the DSC thermogram, even though it was included in the formulation. This result initially suggested strong drug–lipid interactions with crystalline DTX transforming into an amorphous or molecular state. It is known that the solubility, stability, bioavailability, and dissolution rates of drugs vary depending on whether the drugs are crystalline or amorphous.^46−48^ Accordingly, considering that the amorphous form is effective and stable in the active form and in the systemic circulation, observing the drug in an amorphous form in the present DSC analyses was considered to be a positive result. The sharp peak of Dynasan 116 in NLC formulations also collapsed, and a less ordered arrangement was observed, which was attributed to the presence of liquid lipid added to the formulation and the dissolution of docetaxel in lipid structures. Liquid lipid incorporation is thought to cause the delayed crystallization of nanoparticles. These results were found to be compatible with the literature.^49,50^

### *In Vitro* Release

**Figure 4:**
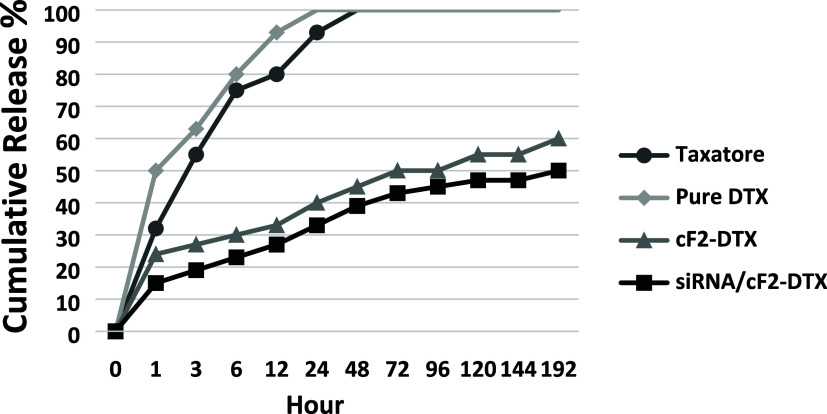
*In vitro* release of pure DTX and DTX from Taxotere, cF2 formulations, and siRNA release of siRNA/cF2-DTX complex at physiological pH 7.4 and temperature 37 ± 0.5 °C.

In the NLC formulation, DTX entrapment efficiency was calculated to be 92.89 ± 0.21% by using the equation. It was reported that in lipid-based drug delivery systems, this percentage depends on the composition and crystalline state of the lipid matrix, and the binding energy of drugs with lipids plays a key role in the successful encapsulation of drugs.^51,52^*In vitro* release analysis was carried out at a physiological blood pH 7.4. Given the results of the release analysis illustrated in Figure 4, regarding the release of DTX from the cF2-DTX formulation within the first hour, it was determined that a burst effect was observed first and then followed by a controlled release. It was observed that, at the end of 192 h, the release percentages of DTX and siRNA from cF2-DTX reached 61.2 and 55.3%, respectively. It is assumed that the initial rapid release of DTX from cF2-DTX is due to DTX accumulating on the outer surface of the particles, and the slower release that occurs later can be attributed to both the strong DTX–lipid interaction and the degradation of the lipid matrix over time. However, another difference among DTX, Taxotere, and cF2-DTX in terms of release properties might be due to the nanostructured lipid nanoparticles having an extended-release function. It is thought that DTX, which is lipophilic and poorly soluble in water, is specifically retained by the lipid core and released through dissolution and diffusion. This result again shows that DTX can be released slowly from nanoparticles with NLC properties and can maintain constant concentration for a relatively longer time.^13,26,53^ The benefit of the extended-release system, as seen in the present study, will be beneficial in reducing the dose and clinical drug administration by maintaining drug concentrations in a therapeutic window for a long period of time. Thus, the prepared formulations suggest the applicability of NLCs as promising drug carrier.

### Cytotoxicity

**Figure 5:**
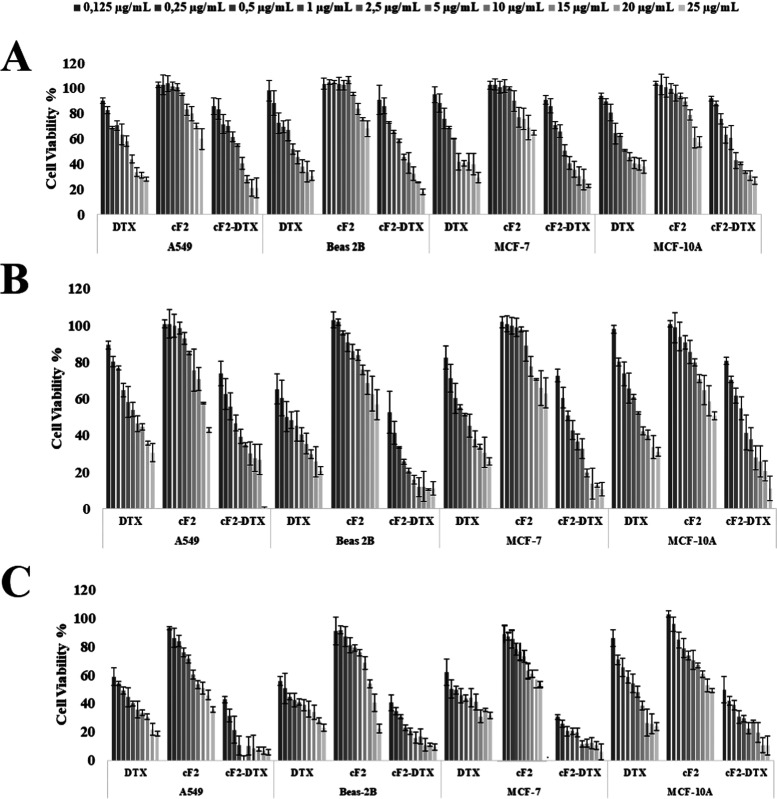
Inhibition results of pure DTX, empty formulation, and DTX-loaded formulations on cells for 24 h (A), 48 h (B), and 72 h (C).

Cytotoxicity studies are used in drug screening and development, examination of the effects of drugs and toxic compounds on cells, evaluation of the effectiveness of mutagenesis and carcinogenesis, production of virus culture and vaccine, as well as obtaining stem cells or pharmaceutical proteins and large-scale production of biological compounds such as vaccines, antibodies, hormones, and therapeutic proteins. The biggest advantage of cell culture for any of these applications is the reproducibility and consistency of the results obtained. Effects on cells and cell responses can be observed directly and, since this effect is direct, it is a rapid type of analysis.^54^ In this study, a comparison was made using healthy and cancer cell lines to evaluate the effects of the commercial agent, DTX, and prepared formulations on the cells. Accordingly, the results are given in Figure 5. Given the results achieved, although dose-dependent cytotoxicity was observed, the IC50 values obtained are given in Table 4.

**Table 4:** IC_50_ Values of DTX, cF2, and cF2-DTX on the Cells at 24, 48, and 72 h (μg/mL) ± SD

	Beas-2B			A549			MCF-10A			MCF-7		
	24 h	48 h	72 h	24 h	48 h	72 h	24 h	48 h	72 h	24 h	48 h	72 h
Taxotere	25.14 ± 5.45	18.69 ± 2.78	5.15 ± 0.33	72.18 ± 4.12	30.27 ± 5.22	9.8 ± 1.55	0.56 ± 0.05	0.08 ± 0.05	0.01 ± 0.05	2.38 ± 0.11	1.91 ± 0.36	0.58 ± 0.69
DTX	5.20 ± 1.35	0.58 ± 0.12	0.26 ± 0.083	6.33 ± 1.56	6.74 ± 1.30	0.09 ± 0.087	5.02 ± 1.78	5.22 ± 1.11	2.71 ± 0.13	3.28 ± 0.39	2.43 ± 0.22	0.27 ± 0.096
cF2	33.85 ± 5.12	27.32 ± 5.32	15.32 ± 1.35	29.9 ± 4.49	22.13 ± 2.33	15.1 ± 1.64	28.7 ± 2.56	25.69 ± 2.44	23.15 ± 3.54	32.35 ± 5.15	31.53 ± 6.56	29.87 ± 5.56
cF2-DTX	3.69 ± 0.32	0.16 ± 0.017	0.101 ± 0.012	5.51 ± 1.03	0.89 ± 0.06	0.08 ± 0.031	3.50 ± 0.187	1.12 ± 0.148	0.113 ± 0.099	2.88 ± 0.011	0.59 ± 0.019	0.076 ± 0.006
siRNA-cF2-DTX	4.02 ± 0.12	0.125 ± 0.05	0.10 ± 0.07	2.15 ± 0.121	0.27 ± 0.015	0.11 ± 0.06	5.01 ± 1.87	0.56 ± 0.081	0.11 ± 0.07	1.23 ± 0.030	0.12 ± 0.012	0.01 ± 0.003

The form of DTX on the market is a product called Taxotere, which contains Tween 80 and ethanol. Since its launch, it has been successfully used solely or in combination with other drugs, particularly in the treatment of advanced breast cancer, non-small-cell lung cancer, ovarian cancer, head and neck cancer, prostate cancer, and stomach cancer. However, there also are concerns that the ethanol contained in the current formulation increases the potential toxicity. For this reason, the development of new formulations is constantly on the agenda.^13^

Comparing the cytotoxicity of the formulations prepared in this study with the current market and DTX application alone, a higher level of cytotoxicity was observed only in A549 cells compared to other cells, and a time- and concentration-dependent increase was observed in the empty formulation in other cells. The cytotoxicity values of the commercial agent that were determined in the present analyses are similar to those cytotoxicity found in the current literature for A549 and MCF-7.^55,56^

No comparison could be made since no values could be found for MCF-10A and Beas-2B. Considering the other results, DTX loaded into the formulation showed a more cytotoxic effect on cells than both the commercial agent and pure DTX (*p* < 0.05). This finding suggests that nanoparticles enable more DTX to be taken into cells. The only drawback at this point is that normal cells were relatively affected. This result indicates that the effectiveness of nanoparticles can be increased by targeting cancer cells in further studies. However, considering the *in vitro* release analyses, it can be seen that DTX is released slowly from the formulations. It can also be seen in the literature that, even with this slow release, lipid-based nanoparticle formulations have a more cytotoxic effect than pure DTX and commercial agents.^53,57,58^

It is thought that this is due to the lipidic structure of the formulation, rapid absorption by the endocytosis mechanism, increased EPR effect, and high permeability, allowing nanoparticles to accumulate inside the cell.^13,59^

In the present study, cytotoxicity evaluation was performed after EphA1, A2, and B3 siRNAs were loaded into the empty formulation (cF2). Attention was paid to ensuring that the formulation dose in the well was 0.25 μg/100 μL, considering the doses used in previous cF2-DTX-loaded formulations. Thus, it was aimed to observe only the effects of siRNAs. In the analyses, 100 ng/100 μL per well was selected for siRNA loading alone, based on previous studies conducted in our laboratory, and then this rate was applied as 50 ng/100 μL and 33.3 ng/100 μL in double and triple combinations, respectively (Figure 4). The aim of this process was to keep the siRNA rate in the well constant. Analyzing the results, it was determined that cell viability decreased to 58.03% with the triple combination of EpHA1/A2/B3 in A549 cells. Since the same effect was observed in the double combination of EpHA2 and B3, it can be stated that siRNAs of EphA2 and EpHB3 alone were more effective on these cells, reducing cell viability to 60.94 and 62.94%, respectively, in 72 h (*p* < 0.05). In dual combinations, cell viability was determined to be 99.89% in EphA1/B3, 60.01% in EpHA2/B3, and 65.88% in EpHA1/A2. Even though it is thought that EphA2 might have played an effective role in the analysis results in Beas-2B cells, cell viability did not fall below 70%, even in triple combinations (*p* > 0.05). Considering the MCF-7 cells, the effect of EpHA2 > EpHB3 > EpHA1 siRNAs was observed in single applications, respectively. It was determined that EphA2/B3 siRNAs reduced cell viability by up to 60% in dual combination applications. Although EphA1 was also determined to have effect on these cells, a statistically significantly higher effect of EpHA2/B3 was observed. In the triple combination, this value decreased to 53.40%. In MCF-10A, cell viability remained above 70% at the 72nd hour and no statistically significant decrease was observed (*p* > 0.05). Although there are many publications in the literature stating that the expression levels of EpH receptors increased in cancer types such as prostate, lung, esophagus, breast, colon, and ovary,^60^ the number of studies on the decrease of these expressions with siRNAs is insufficient. Those studies are generally carried out on cancer cells such as prostate,^61,62^ ovary,^63−67^ glioma,^68^ NSCLC,^69^ breast,^70,71^ squamous-cell carcinoma of the head and neck,^72,73^ and mesothelioma,^74^ where EphA2 is downregulated with siRNA and ASOs in the Eph family. This is because EphA2 is a therapeutic target in many types of cancer, especially since it is associated with poor prognosis, increased metastasis, and decreased survival.^75^ There is an ongoing clinical trial involving DOPC-encapsulated siRNA targeting EphA2 (NCT01591356) in the treatment of patients with advanced or recurrent solid tumors in Phase 1.^76^ Besides that, it was stated in the literature that EphA1, A3, A4, A5, A6, A7, A8, B1, B2, B3, and B4 gain an oncogenic function with their elevated expression in different types of cancer.^77−79^

**Figure 6:**
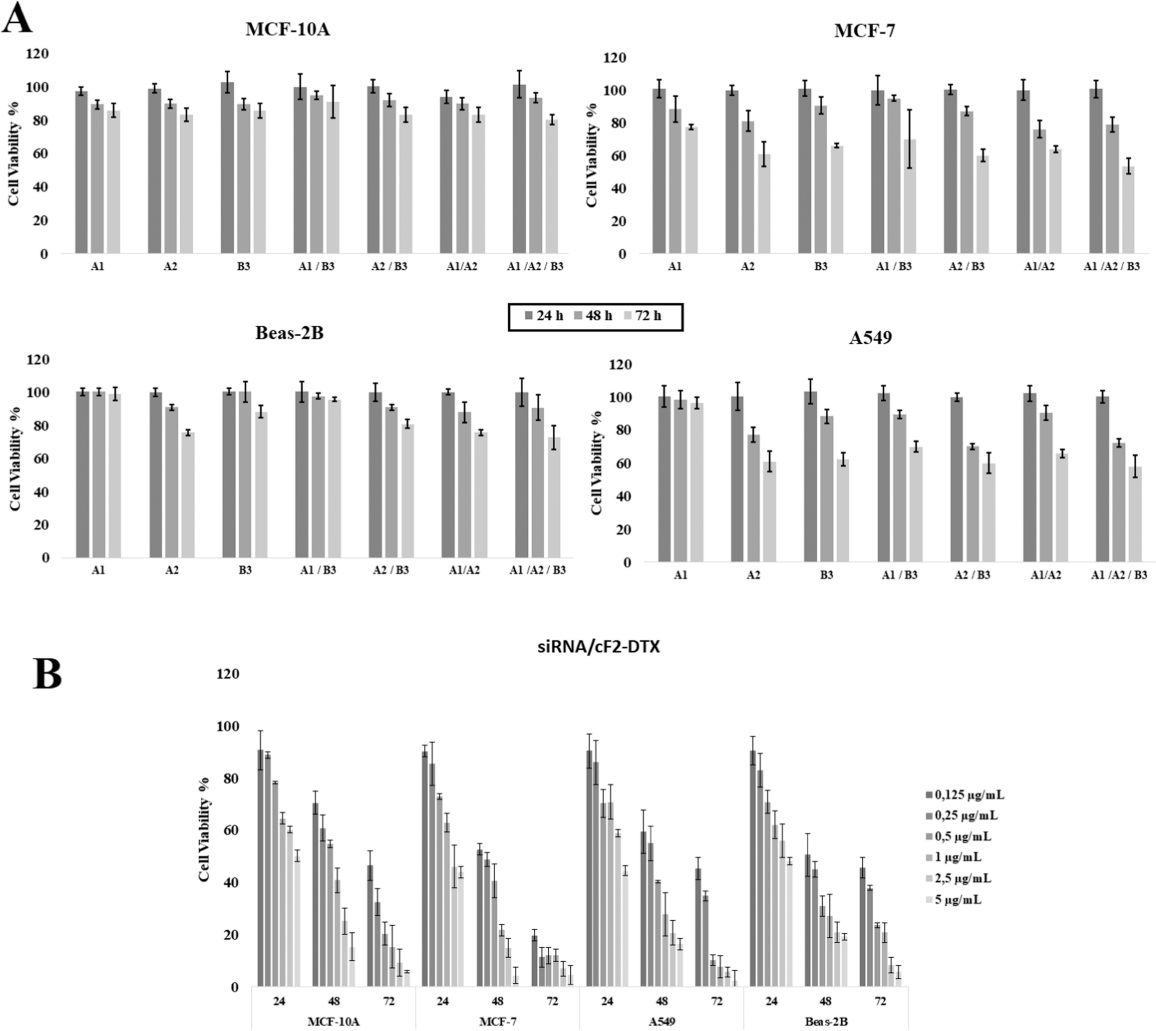
(A) Inhibition results of siRNA EpHA1, A2, and B3 alone and combinations on cells at 24, 48, and 72 h and (B) the effect of siRNA EpHA1/A2/B3 combination on cells after loading into cF2 formulation *The concentration of siRNA to be applied was chosen to be 100 ng/100 μL. In single siRNA applications, the concentration of each siRNA is 100 ng/100 μL; in double combinations, it is 50 ng/100 μL; and in triple combinations, it is 33.3 ng/100 μL each.

Again, in the literature, the studies on Ephs downregulated by RNA interference for these targets are limited. For example, it was stated that EphA3 might increase sensitivity to radiotherapy in head and neck cancers^80^ or it can stop the source of cancer cells in glioblastoma cells,^81^ that EphA7 reduces invasion, migration, and proliferation in human lung cancer cells,^82^ and that EphB4 can be used in cancer treatment by inhibiting its expression in prostate^83^ and breast^84,85^ cancers. For this reason, more studies are needed on the effects of these Ephs. In the present study, the effects of EphA1, EphA2, and EphB3 were evaluated, and the analysis results were presented. Although it was seen in the present study that EphA2 is effective in both types of cancer, it was also determined that EphB3, in addition to EphA2, might be an important marker in these cells. In the literature, a study carried out by Ji et al. in 2011 showed that downregulation of EphB3 with siRNAs inhibited cell proliferation and migration in A549 and H23 cells and suppressed *in vivo* tumor growth and metastasis.^86^ In another study by Stahl et al. in 2013,^87^ it was reported that EpH3 could be an effective target for tumor suppression in U-1810 lung cancer cells. The results reported in some of these studies are in parallel with our results. In addition to all of these results, the effect of siRNAs in combination was studied in the present study for the first time, and it was shown that the combined application in lower amounts was more effective than single applications (Figure 6A).

After the effects of EphA1, EphA2, and EphB3, both alone and in combination, the process was continued with formulations with DTX. After adding EphA1/A2/B3 siRNAs in triple combination to the cF2-DTX formulation, its effect on the cells was evaluated. The siRNA concentration was again adjusted to 33.3 ng/100 μL per well and the prepared complexes were evaluated in the concentration range of 0.125–5 μg/100 μL. The results are given graphically in Figure 6B. In addition, the IC50 values of the complexes are given in Table 4. Examining these results, it was determined that both lower-dose DTX application and the application of siRNAs in combination were much more effective on the cells and that the cells could show activity at lower concentrations starting from the 48th hour.

### Gel Retardation Studies

**Figure 7:**
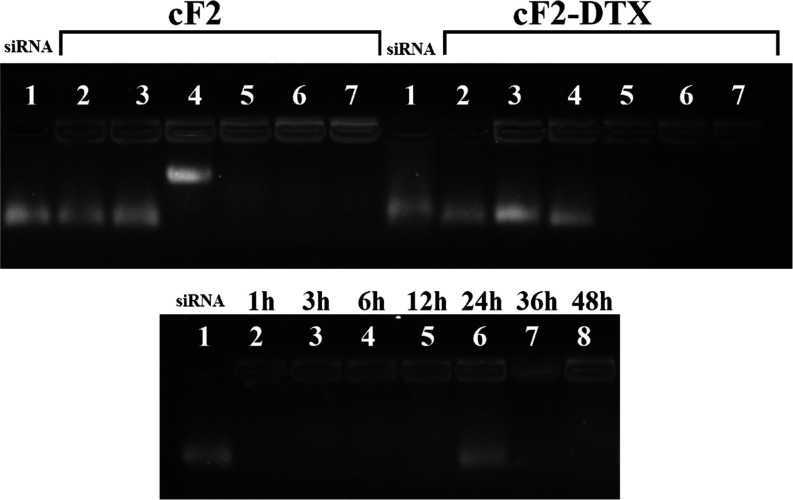
siRNA complex binding efficiency (top) of cF2 and cF2-DTX formulations and protective effect against serum degradation over time (bottom) of the cF2-DTX formulation.

It is an important fact whether the formulations prepared in formulation studies can carry genetic material or protect it against serum nucleases. Particularly since it was planned to be tested in triple combinations rather than alone, the present study investigated how well these siRNAs were retained by the formulation, and their protection against nucleases in the presence of serum was determined by gel retardation. Accordingly, 0.5 μg of each siRNA was taken and subjected to electrostatic interaction with cF2-DTX formulation and cF2 that contains no DTX, which contains 0.5 μL (lane2), 1 μL (lane3), 1.5 μL (lane4), 2 μL (lane5), 2.5 μL (lane6), and 3 μL (lane7), respectively (Figure 7). Then, the electrostatic complexes were loaded onto the gel and the formulations’ capacities to retain genetic material were analyzed under UV. Given the results achieved, it was determined that the 1.5 μL formulation without DTX could keep the siRNAs lightweight, and it released the genetic material since it could not withstand the electrical field anymore. However, it was observed to be able to bind siRNA at higher concentrations. On the other hand, it was observed that DTX-containing formulations could not bind siRNA effectively in the first three concentrations, while it could bind siRNA strongly at 2 μL and higher concentrations. For its protection properties against serum components, 2.5 μL of the formula was first incubated with 0.5 μg siRNA complex and then placed in 50 μL of medium containing 10% FBS. At the end of 1, 3, 6, 12, 24, 36, and 48 h, all of them were loaded into the gel with a loading buffer and the results were analyzed. The results achieved are illustrated in Figure 7 (bottom). Accordingly, it was determined that the formulas started to release siRNA at the 24th hour, and the siRNA was destroyed and lost in the following periods.

### Transfection Studies

**Figure 8:**
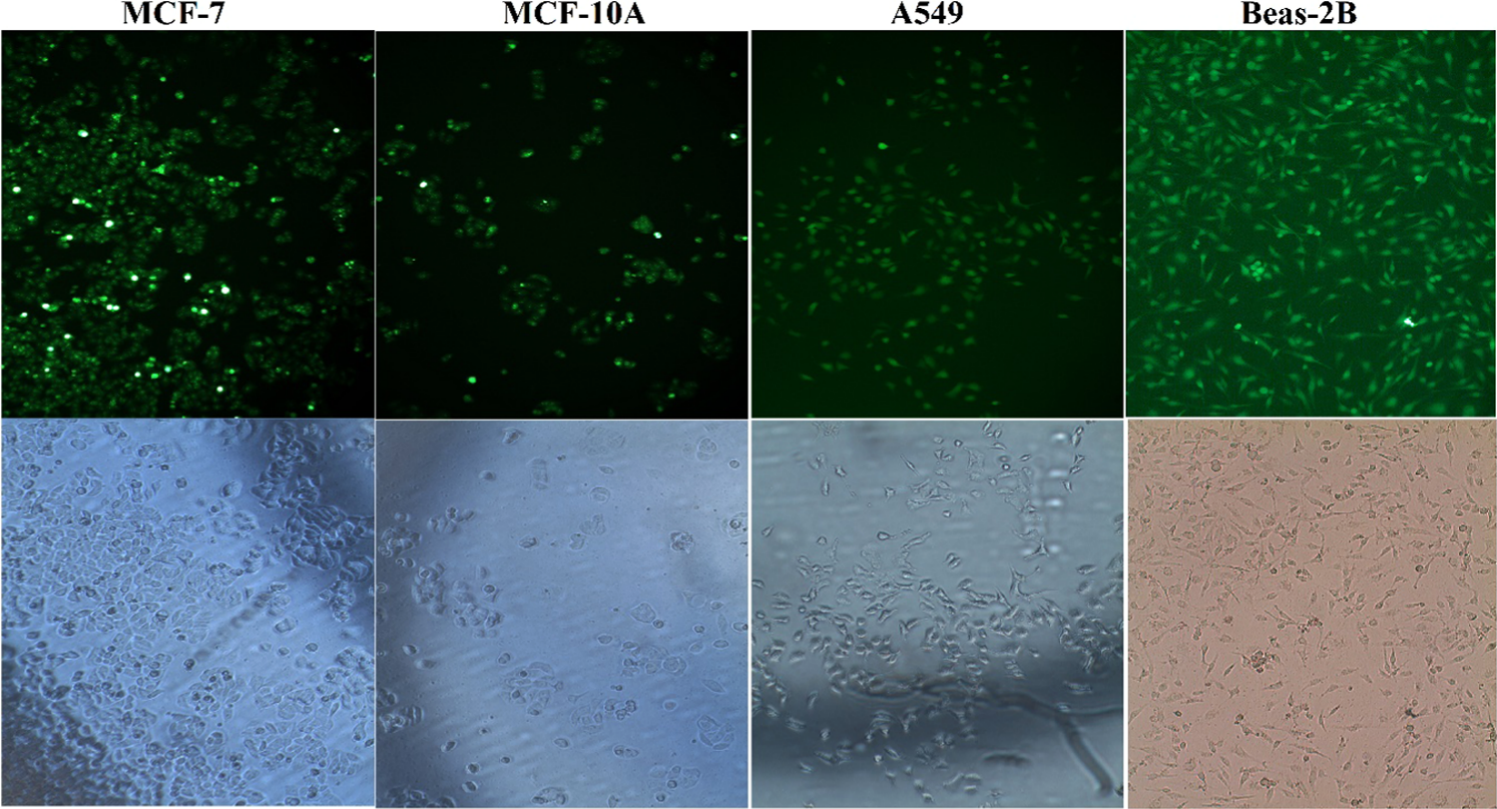
Transfection of cF2-DTX on MCF-7, MCF-10A, A549, and Beas-2B cells.

The transfection study was carried out to determine whether the prepared formulations could be introduced into the cell. Accordingly, although it was observed that the MCF-10 A cell could not reach the desired density in the cells planted before transfection, the transfection efficiency was observed in all cells. It has been analyzed that the prepared formulation delivered siRNA more easily into cells successfully (Figure 8). According to the transfection index results of the analyses performed under the microscope, it was found that 77, 65, 79, and 69% were transfected cells for MCF-7, MCF-10A, A549, and Beas-2B cells, respectively. There were no statistically significant differences between these results (*p* > 0.05).

## Conclusions

There are many siRNA therapeutics being analyzed in various clinical phases. However, the biggest challenge to fully understanding and implementing the potential of RNA-associated therapeutics is site-specific delivery. Large siRNA molecules with anionic properties must overcome various physiological barriers to reach the cytoplasm in target cells. *In vivo* systemic siRNA therapy is hampered by obstacles, such as poor cell uptake, instability under physiological conditions, off-target effects, and possible immunogenicity. For this reason, it is important to either chemically modify siRNAs used in cancer treatment or use them with effective delivery systems. More importantly, optimal combinations of effective delivery systems are thought to lay the foundation for the successful clinical application of siRNAs.^88,89^ For example, there is a lipid carrier of the siRNA therapeutics in the market, and the others consist of chemically defined siRNA bioconjugate (GalNac). Nanoparticles (lipidic, polymeric, inorganic, *etc.*) are often preferred for delivery of nucleic acid molecules. However, regarding the selection of nanoparticles, it should be designed to systematically lead RNAs to target and overcome physiological barriers, provide structural and functional resistance to serum stability, reduce interactions with nontarget cells, increase cell input and endosome escape, resist renal clarity, and create low toxicity and immunogenicity.^88^ In this study, by using DTX as an anticancer drug and siRNA as a nucleic acid therapeutic, it was aimed to develop nanoparticles with a lipidic structure that can be used in cancer treatment. This is because of the small particle dimensions that can facilitate the RNA distribution and cellular intake of lipid nanoparticles and lipid structures that can interact with cell membranes. Many lipids and phospholipids are used to form nanoparticles with a lipid structure. The risk of an unwanted immunogenic reaction to lipids is lower than that of most polymeric materials with higher molecular weights. Most importantly, various lipid nanoparticles continue to be developed for RNA transmission, still liposomes, solid lipid nanoparticles (SLNs), and nanostructure lipid carriers (NLC).^90,91^

In the present study, a carrier system was developed with the solvent evaporation method by using the advantages of NLCs, and DTX and Eph targeted siRNA were successfully loaded onto these particles. The present study investigated the characterization of prepared particles and then their effects on cells in *in vitro* cell culture. The prepared particles were small, had a high ζ potential value, and had high loading efficiency. It was determined that nanoparticles provide higher effectiveness on the cells by using less DTX depending on time. Moreover, with this study, the effect of 3 different Eph siRNA was evaluated simultaneously for the first time by using a lower dose of DTX depending on the cell. The analysis results showed that these particles were more effective than Taxotere and pure DTX. As a result, these nanoparticles, in which DTX and siRNA exhibit a synergistic effect, can be considered an alternative and promising NLC system that can be used in the treatment of diseases.
